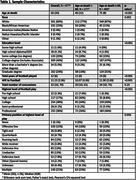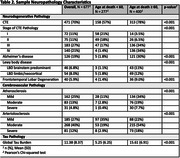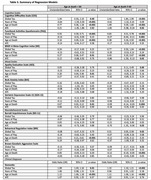# Younger Age of First Exposure to American Football is Associated with Worse Informant‐Reported Clinical Outcomes in Older Age

**DOI:** 10.1002/alz.089438

**Published:** 2025-01-03

**Authors:** Sophia Nosek, Stephanie Gonzalez Gil, Christopher J. Nowinski, Yorghos Tripodis, Brett Martin, Joseph N. Palmisano, Robert C. Cantu, Robert A. Stern, Bertrand Russell Huber, Thor D. Stein, Ann C. McKee, Jesse Mez, Michael L. Alosco, Daniel H. Daneshvar

**Affiliations:** ^1^ Boston University Chronic Traumatic Encephalopathy Center, Boston University Chobanian & Avedisian School of Medicine, Boston, MA USA; ^2^ Concussion Legacy Foundation, Boston, MA USA; ^3^ Boston University School of Public Health, Boston, MA USA; ^4^ Boston University Alzheimer’s Disease Research Center, Boston, MA USA; ^5^ VA Boston Healthcare System, Boston, MA USA; ^6^ VA Bedford Healthcare System, Bedford, MA USA; ^7^ Boston University Chobanian & Avedisian School of Medicine, Boston, MA USA; ^8^ Harvard University School of Medicine, Cambridge, MA USA

## Abstract

**Background:**

Exposure to repetitive head impacts (RHI) is associated with the neurodegenerative tauopathy chronic traumatic encephalopathy (CTE). There is substantial heterogeneity in the clinical presentation of CTE. Younger age of first exposure (AFE) to American football has *not* been associated with odds or severity of CTE. Yet, it has been associated with earlier onset and severity of symptoms, potentially by decreasing resilience to RHI‐related neuropathologies. We examined the association between AFE to football and clinical and neurobehavioral outcomes in deceased football players across the age continuum.

**Methods:**

The sample included 677 male football players who donated their brains to the UNITE Brain Bank. Informants of brain donors completed modified scales assessing donor cognitive function (Cognitive Difficulties Scale [CDS], BRIEF‐A Metacognition Index [MI]), daily function (Functional Activities Questionnaire [FAQ]), mood (Geriatric Depression Scale‐15 [GDS‐15], Beck Anxiety Inventory [BAI] Apathy Evaluation Scale [AES]), and neurobehavioral dysregulation (Barratt Impulsiveness Scale [BIS‐11], BRIEF‐A Behavioral Regulation Index [BRI], Brown‐Goodwin Aggression Scale Adult Sum). Dementia was adjudicated through consensus conferences. Neuropathologists were blinded to clinical history. Global p‐tau burden was calculated by summing semi‐quantitative ratings of p‐tau severity across nine brain regions. Regressions tested the association between AFE and each scale, dementia status, and CTE status. Analyses were performed stratified by age 60 to isolate the effect of neurodegenerative disease resilience adjusting for age, global p‐tau burden, and duration of football play.

**Results:**

Sample characteristics are in **Tables 1‐2**. Most donors played football in college and/or professionally (n = 509, 76%). CTE was the most common neuropathological diagnosis (n = 471, 70%). AFE was not associated with CTE neuropathology. Global tau and age were consistent predictors of the clinical scales (**Table 3**). There were significant associations between AFE and the clinical scales among those older but not younger than age 60. Among those over age 60, younger AFE to football was associated with higher CDS (p = 0.02), BRIEF‐A MI (p = 0.02), and BIS‐11 (p = 0.03) scores.

**Conclusions:**

Younger AFE was associated with worse informant‐reported clinical outcomes in older but not younger deceased football players, independent of tau. Youth exposure to RHI from football might decrease resilience to cope with neuropathologies later in life.